# Study of the Mechanism of Action of Deer Bone Water Extract Fermented by *Lactobacillus reuteri* on Rheumatoid Arthritis-Induced Osteoporosis

**DOI:** 10.3390/nu18111768

**Published:** 2026-05-30

**Authors:** Yingshan Jiang, Xinyu Wei, Jingchen Yan, Yanchao Xing, Yanlu Li, Aoyun Li, Yue Teng, Ting Ren, Weijia Chen, Jianming Li, Ying Zong, Rui Du

**Affiliations:** 1 Sanjiang Laboratory, Jilin Agricultural University, Changchun 130118, China; 2 Laboratory of Production and Product Application of Sika Deer of Jilin Province, Jilin Agricultural University, Changchun 130118, China; 3 College of Agriculture, Yanbian University, Yanji 133002, China

**Keywords:** deer bone, osteoporosis, osteoclasts, *Lactobacillus reuteri*, rheumatoid arthritis

## Abstract

**Background/Objectives:** Osteoporosis (OP) is a frequent complication of rheumatoid arthritis (RA), driven by chronic inflammation and subsequent bone destruction. While deer bone is recognized for its potential bone-health benefits, the therapeutic efficacy of its fermented products on RA-induced OP remains unclear. **Methods:** This study investigated the protective effects of *Lactobacillus reuteri*-fermented deer bone water extract (LR-DBW) against OP in an adjuvant arthritis (AA) rat model. Firstly, LC-MS/MS was employed to analyze the differential peptide profiles between LR-DBW and non-fermented deer bone water extract (DBW). Secondly, experiments such as Micro-CT, histological staining, and Western Blot were conducted to detect the improvement effect of LR-DBW on OP. **Results:** *In vivo*, LR-DBW administration significantly alleviated arthritis symptoms, increased bone mineral density (BMD), and improved bone microarchitecture in AA rats. *In vitro*, LR-DBW inhibited RANKL-induced osteoclastogenesis and actin ring formation in RAW264.7 cells. Mechanistically, LR-DBW inhibits the phosphorylation of ERK, JNK and p38 proteins. **Conclusions:** These research results indicate that one of the mechanisms by which inhibiting osteoclast differentiation into LR-DBW can alleviate osteoporosis caused by rheumatoid arthritis is through down-regulating the phosphorylation expression of ERK, JNK and p38 proteins. This highlights its potential as a functional food component for treating inflammatory bone diseases.

## 1. Introduction

Osteoporosis (OP) is an inflammation-related bone disorder characterized by decreased bone mass and damage to the bone microstructure, resulting in increased bone brittleness and a propensity for fractures, making it a systemic bone disease [1]. The main pathogenesis of osteoporosis is abnormal bone remodeling, which is manifested as an imbalance between osteoclasts (OC) and osteoblasts (OB) [2]. This imbalance may be caused by various factors, especially inflammatory responses [3]. Rheumatoid arthritis (RA) is a typical systemic autoimmune disease [4]. Its main pathological features include joint swelling, pain, and functional impairment, with symmetrical polyarthritis as the main characteristic [5]. It mainly occurs in small joints and leads to severe cartilage and bone destruction [6]. OP is a common complication of RA, and its incidence continues to rise alongside that of RA. This condition significantly increases the risk of fractures and compromises the quality of life in RA patients [7]. The pathogenesis of RA-induced OP is closely linked to inflammatory cytokines that disrupt bone remodeling by promoting osteoclast (OC) activity and suppressing osteoblast (OB) function [8,9]. Therefore, targeting the inflammatory pathways that regulate bone metabolism is a promising strategy for treating RA-induced OP.

The mitogen-activated protein kinase (MAPK) signaling pathway, comprising ERK, JNK, and p38 branches, plays a pivotal role in mediating inflammatory responses and osteoclast differentiation [10,11,12]. Activation of the MAPK pathway by pro-inflammatory cytokines accelerates bone resorption, making it a critical therapeutic target [13]. The commonly used drugs for treating osteoporosis in clinical practice include alendronate sodium, teriparatide, vitamin D calcium supplements, etc. [14,15,16]. However, these drugs can only alleviate the symptoms, and long-term use of them will cause significant side effects. Consequently, there is an urgent need to develop safe, natural alternatives for managing inflammatory bone loss.

Deer bone, a traditional medicinal and nutritional resource, is rich in collagen, peptides, and minerals [17]. In traditional Chinese medicine, it is commonly used to treat conditions such as debility and weak bones, rheumatism and arthralgia, diarrhea and dysentery, abscesses, and skin infections.

Modern studies indicate that deer bone extracts possess anti-inflammatory and immunomodulatory properties [18]. Specifically, experimental data shows that deer bone can effectively improve RA, and deer bone peptide can effectively alleviate osteoporosis induced by ovarian removal in mice [19]. However, currently there are no studies that have clarified the mechanism and effect of the extract from deer bone water in alleviating and treating osteoporosis induced by rheumatoid arthritis. Fermentation is one of the most common processing techniques used in the production of food or medicine [20]. It can alter the structure and composition of proteins, hydrolyze large proteins into bioactive peptides, and have an impact on their functional properties, thereby improving the flavor and quality of food or medicine, enhancing the nutritional value of food or the efficacy of medicine, and promoting absorption by the human body [21,22,23]. *Lactobacillus reuteri* (LR) is mainly found in the intestines of mammals and has strong tolerance to a wide range of pH environments and the digestive system [24]. Current research indicates that Lactobacillus reuteri has potential benefits for various diseases, such as inhibiting inflammatory responses and regulating the body’s immune function [25,26]. Consequently, it represents a promising candidate for fermenting medicinal substrates to enhance their therapeutic potential.

In this study, we systematically evaluated the therapeutic potential of *Lactobacillus reuteri*-fermented deer bone water extract (LR-DBW) against RA-induced OP. We employed LC-MS/MS to characterize the peptide profile of LR-DBW and assessed its efficacy in an adjuvant arthritis (AA) rat model. Our research results indicate that LR-DBW not only improves bone metabolism indicators (serum calcium ions and osteocalcin) but also inhibits the generation of osteoclasts by suppressing the expression of ERK, JNK, and p38 protein phosphorylation. This research provides a scientific basis for the development of deer-bone-based functional foods for the prevention and management of inflammatory osteoporosis.

## 2. Materials and Methods

### 2.1. Materials and Reagents

The deer bones were purchased from Haixia Deer Products Distribution Center in Shuangyang District, Jilin Province, China. The *Lactobacillus reuteri* was purchased from Fenghui Biotechnology Co., Ltd. (Changsha China). The ELISA kits for Ca^2+^, CT, BGP, PTH, ALP, IL-6, extracellular regulated protein kinases (ERK), c-Jun *N*-terminal kinase (JNK) and Matrix metallopeptidase 9 (MMP-9) were purchased from Preferred Biotechnology Co., Ltd. (Shanghai, China). The Xianling Gubao Capsules were purchased from Tongji Hall Pharmaceutical Co., Ltd., of China National Pharmaceutical Group (Guiyang, China). Astragaloside IV was purchased from Nanjing Pu Yi Biotechnology Co., Ltd. (Nanjing, China). Methanol and acetonitrile (HPLC grade) were purchased from Sigma-Aldrich (St. Louis, MO, USA). Formic acid, 3-nitrobenzhydrazide (3-NPH), EDC and pyridine were purchased from Shanghai Aladdin Biotechnology Co., Ltd. (Shanghai, China). All the reference standards were also purchased from Shanghai Aladdin Biotechnology Co., Ltd. (Shanghai, China).

### 2.2. Preparation of DBW and LR-DBW

After the femur of the deer leg was de-fatted with petroleum ether and decalcified with hydrochloric acid, it was extracted using the hot water extraction method. The ratio of deer bone to distilled water was 1:10, the temperature was 100 °C, and the time was 3 h per extraction, with a total of three extractions. The combined extract solution was concentrated and then freeze-dried to obtain a completely water-soluble powder of deer bone extract. By optimizing key parameters such as inoculation volume, temperature, time, and liquid-to-solid ratio, the influence of LR on the protein content of the deer bone water extract was evaluated. Finally, the optimal conditions were determined as follows: LR inoculation volume of 5%, temperature of 37 °C, fermentation time of 20 h, and liquid-to-solid ratio of 30:1. After the fermentation process was completed, the fermentation product was centrifuged at 3000 rmp for 15 min at 4 °C to remove *Lactobacillus reuteri*. The supernatant was then concentrated and freeze-dried to obtain LR-DBW powder. The total protein concentration was determined using the BCA assay kit (Shanghai Biyuntian Biotechnology Co., Ltd., Shanghai, China), and the experiment was repeated three times to verify the results. The obtained LR-DBW showed excellent stability.

### 2.3. Determination of Protein Composition in Unlabeled Proteomics

#### 2.3.1. LC-MS/MS Analysis

The peptide segments were desalted using a C18 column and then freeze-dried. The LRH samples were separated using an HPLC system with two buffers. Buffer A was a formic acid aqueous solution, and Buffer B was a formic acid acetonitrile aqueous solution (with acetonitrile at 80%). After the chromatographic column was equilibrated with 100% A solution, the samples were injected into a mass spectrometry pre-column (C18 3 µm 100 µm × 20 mm, Thermo Fisher Scientific, Waltham, MA, USA) and then separated through an analytical column (C18 1.9 µm 150 µm × 120 mm, Thermo Fisher Scientific).

After the samples were separated by capillary HPLC, they were analyzed using a Q Exactive HF-X mass spectrometer (Thermo Fisher Scientific, Waltham, MA, USA). The parameter settings were as follows: analysis time 75 min, detection mode positive ion, parent ion scan range 300–1400 *m*/*z*, first-stage mass spectrometry resolution set to 120,000, AGC target 5 × 10^5^, maximum injection time (IT) 50 ms, dynamic exclusion time 20 s. The MS2 activation method used HCD (high-energy collision dissociation), with the isolation window set to 1.6 *m*/*z*, micro-scanning set to single scan, the second maximum injection time (IT) 35 ms, and normalized collision energy adjusted to 30 eV.

#### 2.3.2. Quantitative Analysis of Differential Proteins, GO Functional Annotation, and KEGG Pathway Annotation

Under the threshold condition of a 2-fold difference (FC = fold change), the differentially expressed proteins were screened. If 0.5 < FC < 2, it is considered that the expression level has no significant change; if FC ≤ 0.5, it indicates down-regulation; if FC ≥ 2, it indicates up-regulation. All the differentially expressed proteins were annotated for their GO functions using the EBI database resources and InterProScan (version 5.52-86.0) software. The number of differentially expressed proteins was statistically analyzed at the GO secondary functional annotation level. The proteins were annotated and analyzed through the KEGG pathway database.

### 2.4. Analysis of Amino Acid Composition

Take 0.02 to 0.03 g of the sample, add 50% concentrated hydrochloric acid (HCl) to it, then introduce nitrogen gas into the nitrogenation device to protect the sample from oxidation. Place the treated sample in a 111 °C oven for a digestion process of up to 22 h. After the digestion is completed, filter, increase the volume to 50 mL, take 700 μL of the water-permeable filter membrane and transfer it into a small bottle, and use the automatic amino acid analyzer (Hitachi, Tokyo, Japan) for analysis.

### 2.5. Grouping and Administration in Animal Experiments

Eighty SPF-grade female Wistar rats, weighing 160–180 g (approximately 6 weeks old), were purchased from Yisi Laboratory Animal Technology Co., Ltd. (Changchun, China), with the license number SCXK (JI) 2023-0002. The animal welfare and ethics committee of Jilin Agricultural University (Ethical Review Approval Number: 20211011003) approved all the animal experiments. The experimental animals were housed under a temperature of 25 ± 2 °C and a 12:12 h light–dark cycle. All the animal procedures were in accordance with the 1986 UK Animal (Scientific Procedures) Act, the EU Animal Protection Directive 2010/63/EU, and the NIH Laboratory Animal Care and Use Guidelines (NIH Publication No. 8023, revised in 1978). Before the start of the study, the animals were allowed to adapt to the new environment for one week, and all the rats had free access to food and water. One week after adaptation, they were randomly divided into 8 groups (10 rats per group). The adjuvant-type rat arthritis model (AA) was established: For the AA group rats, 0.1 mL of complete Freund’s adjuvant was subcutaneously injected into the foot of the right hind leg, while the blank control group received the same amount of normal saline at the same site. Seven days later, 0.1 mL of incomplete Freund’s adjuvant was injected into the same site of the AA group rats, and the blank control group received the same amount of normal saline. For administration, the rats were then randomly divided into 8 groups (n = 10):

1. Blank control group (BG): Normal saline (dose: 2 mL);
2. Model group (MG): AA + normal saline (dose: 2 mL);
3. High-dose group of deer bone water extract (WH): AA + DBW (dose: 0.16 g/kg);
4. Low-dose group of deer bone water extract (WL): AA + DBW (dose: 0.04 g/kg);
5. High-dose group of fermented deer bone water extract (LRH): AA + LR-DBW (dose: 0.16 g/kg);
6. Low-dose group of fermented deer bone water extract (LRL): AA + LR-DBW (dose: 0.04 g/kg);
7. Positive drug group Xianling Guobao Capsules (XL): AA + Xianling Guobao Capsules (dose: 1 g/kg);
8. Inhibitor group (AST): AA + Astragaloside IV + LR-DBW (dose: 0.16 g/kg LR-DBW, 0.2 g/kg Astragaloside IV).

Each treatment group was given the corresponding treatment at the specified dosage by intragastric administration. The degree of joint swelling in each group was observed. Approximately 24 h after the last administration at the end of the 21-day treatment course, the rats were euthanized by intraperitoneal injection of pentobarbital sodium (200 mg/kg). Subsequently, femoral and tibial samples were collected and stored at −80 °C for subsequent analysis.

### 2.6. Toe Volume, Arthritis Score and Weight Change

Starting from the first day after vaccination, the toe volume of each group of rats was measured and recorded using a toe volume meter (Nanjing Calvin Biotechnology Co., Ltd., Nanjing, China). Fixed marks were made on the ankle joints of each rat, and the ankle joints were extended so that the marks were aligned with the water surface of the instrument. Then, the instrument readings were recorded. On days 0, 7, 14, 21, 28, 35 and 42 after modeling, the range of toe volume, arthritis score (Table 1), and weight changes of each group of rats were evaluated and recorded.

### 2.7. Serum Biochemical Index Testing

After the last administration, blood was collected from the abdominal aorta of the rats for anesthesia and blood sampling. The collected whole blood was left to stand, then centrifuged at 2000 r/min for 10 min to extract the serum. The serum was frozen at −20 °C for storage. The sample was thawed at room temperature and homogenized. The supernatant was then taken for measurement. Using the enzyme-linked immunosorbent assay method, according to the operation steps in the kit manual, the levels of Ca^2+^, Calcitonin (CT), Bone-γ-Carboxyglutamic Acid-Containing protein (BGP), Parathyroid Hormone (PTH), alkaline phosphatase (ALP) and IL-6 in the rat serum were detected.

### 2.8. Micro-CT Examination

After the death of each group of rats, their femurs and tibias were quickly extracted, the attached soft tissues were removed, and then the bone tissues were placed in a 4% polyformaldehyde solution for fixation for 24–48 h. The femoral samples of each group were scanned using micro-computed tomography-50 (Scanco Medical, Brüttisellen, Switzerland), with each layer being 10 μm. The femoral long axis was placed parallel to the sample tube long axis in a 25 mm diameter sample tube. The scanning parameters were a voltage of 70 KVp, a current of 200 μA, and a resolution of 48.2 μm. After the scanning was completed, three-dimensional reconstruction and bone volume statistics analysis were performed using the relevant analysis software of the equipment. The threshold was set between 212 and 1000, and parameters such as the number of bone trabeculae (Tb. N), the thickness of bone trabeculae (Tb. Th), the spacing of bone trabeculae (Tb. Sp), and the ratio of bone surface area to bone volume (BS/BV) were analyzed.

### 2.9. H&E and TRAP Staining

After the administration was completed, the distal 1/3 of the femurs of each group of rats was taken, the attached tissues were removed, and then the bone tissues were fixed in 4% paraformaldehyde solution, embedded, sectioned, stained, and made into non-decalcified bone sections. After sectioning, hematoxylin and eosin staining was performed, along with dehydration and mounting, and the pathological changes of the femoral ends of each group of rats were observed. Another part of the sections was stained with TRAP staining solution for 1 h, stained with hematoxylin for 2 min, dehydrated, transparentified with tree gum, and mounted for observation of the growth of osteoclasts under an optical microscope.

### 2.10. Western Blot Is Used to Detect the Expression of Factors Related to Bone Tissue

Three femoral samples were randomly selected from each group of rats. After removing the femurs, they were added to the lysis buffer (the lysis buffer was mixed with proteinase inhibitors at a ratio of 100:1 before use) and then thoroughly ground using a tissue homogenizer(NewZhi Biotechnology Co., Ltd., Ningbo, China). The mixture was placed on ice and left to stand for 15 min. It was then centrifuged at 12,000 rmp/min for 15 min at 4 °C. The supernatant was collected to obtain the total protein of the bone tissue. The total protein in each group of bone tissue was quantified using the BCA kit. Then, loading buffer was added, and the mixture was boiled in boiling water for 15 min. Finally, protein samples were obtained. The samples were separated by SDS-PAGE, then transferred to a PVDF membrane and incubated with appropriate primary antibodies (ERK: 1:5000; JNK: 1:1000; p38: 1:5000; GAPDH: 1:100,000; p-ERK: 1:1000; p-JNK: 1:1000; p-p38: 1:1000; Goat Anti-Rabbit IgG:1:5000; all the antibodies were purchased from StarTech Biotechnology Co., Ltd., Hangzhou, China) for 2 h at 4 °C. After incubation, the sample was washed 5 times with TBST for 10 min each time, followed by incubation with the secondary antibody for 2 h. The secondary antibody was removed, and the sample was washed 5 times in a shaker using TBST for 10 min each time. The ECL developing solution was prepared in a 1:1 ratio. It was allowed to stand in the dark for 30 s, then it was placed on the instrument for imaging. It was analyzed using Image J. Each sample was subjected to three technical replicate tests. The results were expressed as the mean ± standard deviation.

### 2.11. Preparation of Drug-Containing Serum

Thirty SPF-grade female Wistar rats, weighing 160–180 g (approximately 6 weeks old), were purchased from Yisi Laboratory Animal Technology Co., Ltd. (Changchun, China), with the license number SCXK (JI) 2023-0002. The animal welfare and ethics committee of Jilin Agricultural University (ethics approval number: 20211011003) approved all the animal experiments. The experimental animals were housed under a temperature of 25 ± 2 °C and a 12:12 h light–dark cycle. All the animal procedures were in accordance with the 1986 UK Animal (Scientific Procedures) Act, the EU Animal Protection Directive 2010/63/EU, and the NIH Laboratory Animal Care and Use Guidelines (NIH Publication No. 8023, revised in 1978). Before the start of the study, the animals were allowed to adapt to the new environment for one week, and all the rats had free access to food and water. One week after adaptation, they were randomly divided into 5 groups (6 rats per group):

1. Blank control group (BG): Normal saline (dose: 2 mL);
2. High-dose group of deer bone water extract (WH): DBW (dose: 0.16 g/kg);
3. Low-dose group of deer bone water extract (WL): DBW (dose: 0.04 g/kg);
4. High-dose group of fermented deer bone water extract (LRH): LR-DBW (dose: 0.16 g/kg);
5. Low-dose group of fermented deer bone water extract (LRL): LR-DBW (dose: 0.04 g/kg).

The drug was administered once a day for 7 consecutive days according to the prescribed dosage. Approximately 2 h after the last administration, the rats were euthanized by intraperitoneal injection of pentobarbital sodium (200 mg/kg), and blood was collected from the abdominal aorta of the rats. The whole blood was centrifuged at 4 °C, 12,000 r/min for 15 min, and the supernatant was taken and aliquoted. It was then incubated in a water bath at 56 °C for 30 min to inactivate the blood, and the serum was filtered through a 0.22 μm microporous membrane. The serum was aliquoted into centrifuge tubes and stored at −80 °C for future use.

### 2.12. Detection of the Effect of LR-DBW on the Survival Status of Raw264.7 Cells Using the CCK-8 Method

To eliminate the influence of the toxic effects of the drug on cell activity, the cytotoxicity of different concentrations of LR-DBW on Raw264.7 cells was observed using the CCK-8 assay (Biyun Tian Biotechnology Co., Ltd., Shanghai, China). RAW264.7 cells were taken, and cell suspension was prepared using DMEM high-glucose medium (Senopu Biomedical Technology Co., Ltd., Beijing, China) containing 10% fetal bovine serum. Approximately 100 μL of the cell suspension was inoculated into each well of a 96-well plate, with 10,000 cells per well. The cells were cultured at 37 °C and 5% CO_2_ for 12 h to adhere to the plate. After adhesion, the culture medium was replaced with the culture medium containing different concentrations (5%, 10%, 15%, 20%, 25%, 30%, 35%, 40%) of LRH-containing serum. The group without added serum was set as the fetal bovine serum control group (FCS). Each sample was set with 3 replicates. The cells were further cultured under the same conditions for 24 h. A total of 20 μL of the CCK-8 solution was added to each well, and the cells were cultured for another 4 h. The absorbance of the cells at 450 nm was measured using an enzyme reader (Bertong Instrument Co., Ltd., Burlington, VT, USA), and the cell survival rate was calculated.

### 2.13. Induce RAW264.7 Cells to Differentiate into Osteoclasts

RAW264.7 cells in the logarithmic growth phase were inoculated into 96-well plates for cultivation. The culture medium was α-MEM medium containing 10% fetal bovine serum. When the cells adhered to the plate at 90%, RANKL (100 ng/mL) was added. According to the results of the CCK-8 experiment, the optimal drug concentration was 25% drug-containing serum, and osteoclasts were induced at this concentration. The groups were as follows:

1. RAW264.7 + RANKL;
2. RAW264.7 + RANKL + 25% BG group with drug-containing serum;
3. RAW264.7 + RANKL + 25% WH group with drug-containing serum;
4. RAW264.7 + RANKL + 25% WL group with drug-containing serum;
5. RAW264.7 + RANKL + 25% LRH group with drug-containing serum;
6. RAW264.7 + RANKL + 25% LRL group with drug-containing serum.

The culture medium the cells were suspended in was changed every 2 days, and TRAP staining was performed 7 days after induction.

### 2.14. F-Actin Ring Formation

Take RAW264.7 cells that are in the logarithmic growth phase, induce them to differentiate into osteoclasts, allow the cells to culture overnight, and make their density reach 50–60%. After washing, fix the cells with 4% paraformaldehyde for 10–30 min. After fixation, wash the cells with PBS 2–3 times, each time for 10 min, at room temperature. Treat the cells with 0.5% Triton X-100 solution for 30 min for permeabilization. After permeabilization, seal with 1% BSA for 30 min. After sealing, add the rhodamine-labeled chytrid peptide staining solution for 30 min under light protection. After staining, add the DAPI solution containing the mounting agent and observe with a fluorescence microscope (Olympus Corporation, Tokyo, Japan). Use Image Pro Plus 6.0 software to calculate the number and area of the actin rings formed by osteoclasts in each well.

### 2.15. Western Blot Detection of the Effect of LR-DBW on the Expression of Proteins Related to the MAPK Signaling Pathway

RAW264.7 cells were seeded at a density of 5000 cells per well in a 6-well plate and incubated in a constant temperature incubator with 5% CO_2_ for 12 h. The cells were then grouped as described in Section 2.12, and both the control group and the drug group were subjected to 4 h starvation treatment, followed by stimulation with 25% serum containing the drug. After 4 h, 100 ng/mL RANKL was added to the drug group and FCS group in each group, and the stimulation time was 0 min, 15 min, 30 min, and 60 min. When the time reached 60 min, the cells were lysed and the total protein was extracted. The protein was quantified using the BCA method, and all the samples were diluted to the same concentration. The Western blot experiment was performed according to the experimental method 2.9, and three technical repetitions were performed.

### 2.16. Statistical Analysis

The experimental data are expressed with mean standard deviation (Mean ± SD), and the data were statistically analyzed using SPSS Statistics 26. The mean values between the samples were compared using one-way analysis of variance (ANOVA) followed, by Tukey’s post hoc test for group comparisons, and the image was analyzed using Image J 2.14.0 software, with *p* < 0.05 being statistically significant.

## 3. Results

### 3.1. LC-MS/MS

#### 3.1.1. Identification of Proteins in LR-DBW

**Figure 1:**
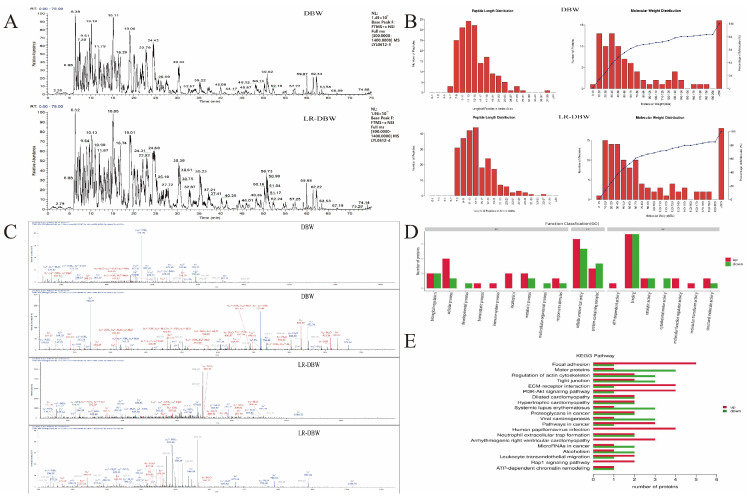
Label-free quantitative proteomics analysis. (**A**) Mass spectrum Basepeak of DBW and LR-DBW; (**B**) the lengths of DBW and LR-DBW peptides, as well as the molecular weights of the proteins; (**C**) in DBW and LR-DBW, two types of peptides with the highest content were identified; (**D**) functional annotation of differential protein GO; (**E**) KEGG Differentially Expressed Protein Pathway Annotation.

As shown in Figure 1A, 118 proteins and 248 peptide segments were identified from LR-DBW, while 97 proteins and 197 peptide segments were identified from DBW. After fermentation, the number of proteins increased by 21.6%, and the number of peptide segments increased by 25.9%. The fermentation effect of *Lactobacillus reuteri* significantly enriched the active protein/peptide component in the aqueous extract of deer bone, significantly enhancing the diversity of the product components. The distribution of peptide segment lengths in the two sample groups is shown in Figure 1B. LR-DBW is mainly composed of peptide segments with 7 to 37 peptide bonds, while DBW is mainly composed of peptide segments with 5 to 39 peptide bonds. Compared with DBW, the peptide segment length distribution of LR -DBW is more concentrated, and some short, small molecule peptide segments have been removed, retaining more medium-length active peptide segments. These medium-length peptide segments are more easily absorbed by the body and can stably exert biological activity, avoiding the defects of short peptides being easily degraded and long peptides having low absorption efficiency. Figure 1C shows the two peptide segments with the strongest signals in LR -DBW and DBW. The sequences of the two peptide segments in LR -DBW are VGWEQLLTTIAR and IEVLEEELR. The sequences of the two peptide segments in DBW are ADFAEVTKIVTDLTK and RPCFSALTLDETYVPK. The proteolysis of *Lactobacillus reuteri* causes the large molecular proteins in deer bone to undergo specific cleavage, generating novel characteristic peptide segments that do not exist in DBW. These peptide segments may be the components responsible for the biological effects of LR-DBW. Through LC-MS/MS comparative analysis, it was found that LR -DBW contains more types of small-molecule peptides and higher content than DBW, and there are specific peptide segments that are specific to the fermentation process. These components may serve as the basis for the inhibitory effect of LR-DBW on osteoclast differentiation and the improvement of bone metabolism and also explain the significant enhancement of its biological activity.

#### 3.1.2. GO Function Annotation and KEGG Pathway Annotation

LR-DBW contained 49 up-regulated proteins and 39 down-regulated proteins in comparison to DBW, according to a quantitative analysis of the differential proteins in the two sample groups. Biological regulation, cell processes, developmental processes, homeostasis processes, immune system processes, localization, metabolic processes, physiological processes of multicellular organisms, response to stimuli, cellular structural entities, protein-containing complexes, ATP-dependent activities, binding, catalytic activities, cytoskeletal movement activities, molecular function regulatory activities, molecular converter activities, and structural molecular activities are among the eighteen biological processes covered by the GO functional annotations of the differential proteins displayed in Figure 1D. At the molecular function level, it primarily concentrates on binding activity, catalytic activity, cytoskeletal movement activity, and molecular function regulatory activity. The core enrichment is found in processes that are closely associated with the pathological mechanism of RA-OP, including cellular processes, metabolic processes, immune system processes, and stress responses. These mechanisms directly participate in regulating inflammatory factors and the proliferation and differentiation of osteoclasts, as well as the process of bone resorption. They may constitute the main functional basis for the preventive effect of LR-DBW on secondary osteoporosis.

The findings of the KEGG pathway annotation for the differential proteins are shown in Figure 1E. These include focal adhesion, actin, actin cytoskeleton regulation, tight junction, ECM-receptor interaction, the PI3K–Akt signaling pathway, dilated cardiomyopathy, hypertrophic cardiomyopathy, systemic lupus erythematosus, proteoglycans in cancer cells, viral carcinogenesis, cancer pathways, human papillomavirus infection, neutrophil extracellular trap formation, arrhythmogenic right ventricular cardiomyopathy, alcoholism, leukocyte transendothelial migration, ATP-dependent chromatin remodeling. The key pathways include focal adhesions, actin cytoskeleton control, ECM-receptor interaction, the PI3K–Akt signaling pathway, and the Rap1 signaling pathway and are where the differential proteins are mostly concentrated. The ECM-receptor interaction pathway is involved in the regulation of bone microenvironment homeostasis. The actin cytoskeleton regulation pathway directly promotes the formation of actin rings in osteoclasts (the actin rings are the structural basis for osteoclasts to perform bone resorption functions), and the PI3K–Akt signaling pathway and the MAPK signaling pathway have cross-regulatory effects with specific albumins, which can synergistically inhibit the activation and differentiation of osteoclasts. However, the protein components of DBW did not show specific enrichment in these pathways, which might be the fundamental reason why LR-DBW has a greater advantage over DBW in improving bone microstructure and enhancing anti-resorption effects.

### 3.2. Analysis Results of Amino Acids

**Figure 2:**
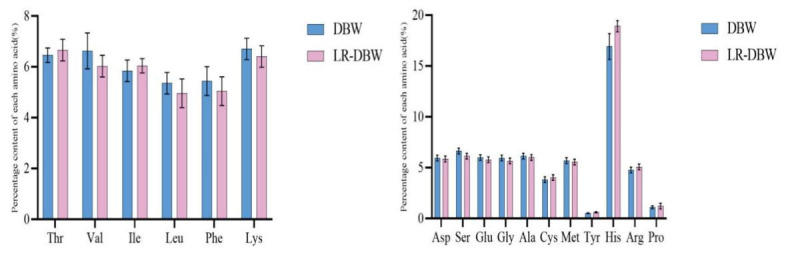
Percentage content of amino acids in LR-DBW and DBW.

The amino acid composition of the LR-DBW and DBW samples was analyzed, and the results are shown in Figure 2. A total of 17 amino acids were detected in LR-DBW, among which histidine (18.917%) had the highest content. The analysis results indicate that not only were various non-essential amino acids abundant in LR-DBW, but it also contained many amino acids that could meet the daily nutritional needs of the human body, support physiological metabolic processes, and have positive effects on promoting bone health growth and development. For example, glycine and proline were included. Therefore, as a nutritional source, LR-DBW demonstrates potential beneficial impacts on the development of human health food raw materials.

### 3.3. General Behavioral Observation of Rats

**Figure 3:**
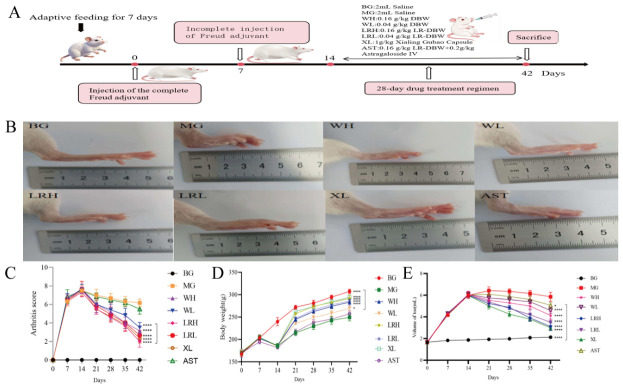
Model establishment and general observation of rats (n = 10). (**A**) Timeline of animal experiments; (**B**) the overall observation results after treatment for all the groups; (**C**) arthritis score; (**D**) body weight change; (**E**) change in toe volume. (Compared with the BG group: compared with the MG group: * *p* < 0.05, **** *p* < 0.0001).

The experiment first established a model of osteoporosis induced by rheumatoid arthritis (Figure 3A). The volume of the rats’ toes, changes in body weight, and arthritis scores of each group were analyzed (Figure 3C–E). Through observation, it was found that compared with the MG group, the degree of joint swelling in the treatment group was significantly reduced (Figure 3B). The experimental results show that after all the treatment groups were given the drug treatment, the toe volume was significantly improved compared with the MG group (*p* < 0.05), the swelling degree was relieved, and the trend was consistent with the XL group. The changes in body weight among the groups might be due to the limitation of movement caused by swelling and pain, thereby reducing food intake and weight loss. Except for the AST group, the arthritis score index of the other treatment groups was significantly lower compared with the MG group (*p* < 0.05), and the trend was consistent with the XL group.

### 3.4. The Effects of LR-DBW on Serum Indicators of Rats

**Figure 4:**
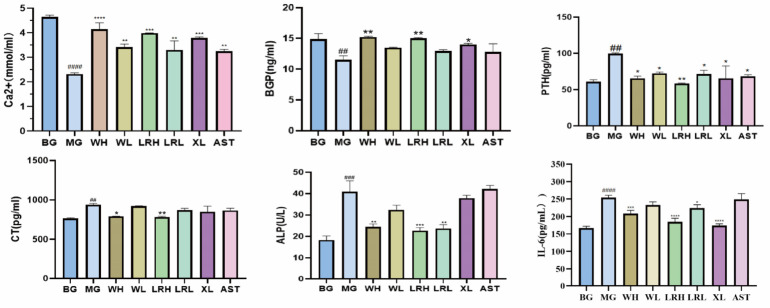
The influence of LR-DBW on serum indicators of rats (n = 10). (Compared with the BG group: ## *p* < 0.01, ### *p* < 0.001, #### *p* < 0.0001; compared with the MG group: * *p* < 0.05, ** *p* < 0.01, *** *p* < 0.001, **** *p* < 0.0001).

The experimental results show that the levels of Ca^2+^ and BGP in the serum of rats in the MG group were significantly decreased, while the levels of CT, ALP and PTH were significantly increased, showing significant differences compared with the BG group (*p* < 0.05). Combined with the degree of joint swelling and the arthritis score of the rats, it indicates that the osteoporosis model induced by RA has been successfully established. After drug treatment, compared with the MG group, the levels of Ca^2+^ and BGP in the serum of rats in the WH group and LRH group were significantly increased (*p* < 0.05), while the levels of CT, ALP and PTH were significantly decreased (*p* < 0.05) (Figure 4), consistent with the trend of the XL group. The improvement of these serum indicators further confirmed the positive regulatory effect of LRH on the calcium and phosphorus metabolism and bone metabolism balance in rats with RA-induced osteoporosis. Compared with the MG group, the level of Ca^2+^ in the AST group was significantly increased, and the level of PTH was significantly decreased (*p* < 0.05).

### 3.5. The Effect of LR-DBW on the Bone Microstructure of Rats

**Figure 5:**
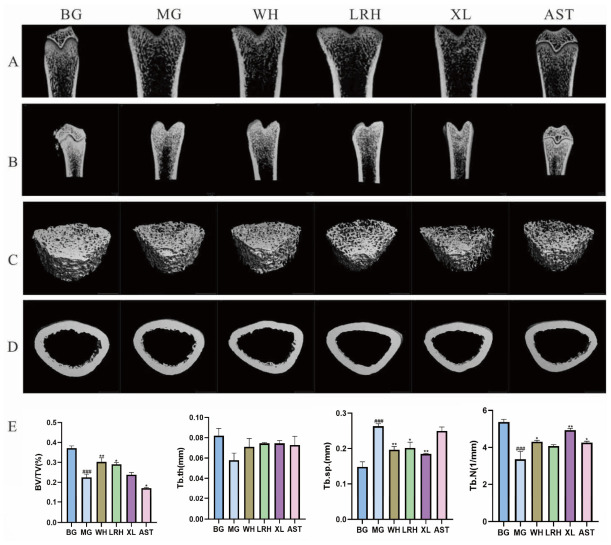
Effects of LR-DBW on the bone microstructure of rats (n = 10). (**A**) Two-dimensional cross-sectional image of the rat femur; (**B**) three-dimensional cross-sectional image of the rat femur; (**C**) 3D trabecular bone image of the rat femur; (**D**) 3D cortical bone image of the rat; (**E**) bone histological parameters. (Compared with the BG group: ### *p* < 0.001; Compared with the MG group: * *p* < 0.05, ** *p* < 0.01).

We conducted imaging on the femurs of different treatment groups and used 2D/3D reconstruction techniques of micro-computed tomography (Micro-CT) to examine the changes in their microstructure. The proximal bone trabeculae in the MG group were significantly sparser than those in the BG group (Figure 5A,B), with a significant reduction in the number of trabeculae and bone volume/total volume (BV/TV), and a significantly increased degree of trabecular separation (*p* < 0.05). However, the thickness of the trabeculae did not change significantly. The arrangement of trabeculae in the LRH group was denser than that in the MG group, with a significant increase in the number of trabeculae and bone volume/total volume (BV/TV) (*p* < 0.05), and a significantly decreased degree of trabecular separation (*p* < 0.05). However, the thickness of the trabeculae did not change significantly (Figure 5C), which was consistent with the trend of the XL group.

### 3.6. Histological Staining Results

#### 3.6.1. H&E Staining Results

**Figure 6:**
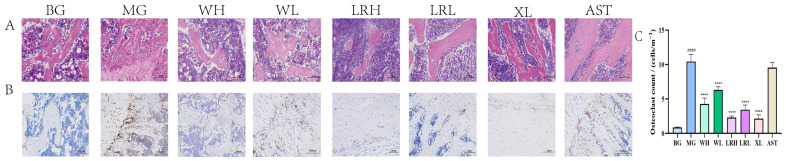
Histological staining results. (**A**) H&E staining results; (**B**) TRAP staining results (n = 10). (Note: The red arrow points to the osteoclast); (**C**) Comparison of the number of osteoclasts in the femoral tissues of each group of rats. (Compared with the BG group: #### *p* < 0.0001; compared with the MG group: **** *p* < 0.0001).

To observe the effect of LR-DBW on bone loss in rats, we performed hematoxylin–eosin staining on the femoral tissues of the rats. The results show that the bone trabeculae in the BG group were thicker and darker in color, with a relatively complete structure, and there were a large number of hematopoietic red blood cells in the bone marrow cavity. In contrast, the bone trabeculae in the MG group were significantly sparse and fragmented, the area was decreased, and the number of hematopoietic red blood cells in the bone marrow cavity also decreased. After treatment, the bone trabeculae in each treatment group were arranged more closely, fractures decreased, and the number of red blood cells in the bone marrow cavity significantly increased (Figure 6A), which was consistent with the XL group. The results indicate that fermented deer bone water extract can effectively alleviate the phenomenon of bone loss in rats.

#### 3.6.2. TRAP Staining Results

To observe the effect of LR-DBW on the number of osteoclasts in rats, we performed TRAP staining on the femoral tissues of the rats (Figure 6B). The results show that compared with the BG group, the number of osteoclasts in the MG group significantly increased. After drug treatment, the number of osteoclasts decreased significantly, and the trend was consistent with that of the XL group. Compared with the MG group, the number of osteoclasts in the AST group did not show a significant decrease.

### 3.7. Detection of the Expression Levels of Related Proteins in Bone Tissue by Western Blot Method

**Figure 7:**
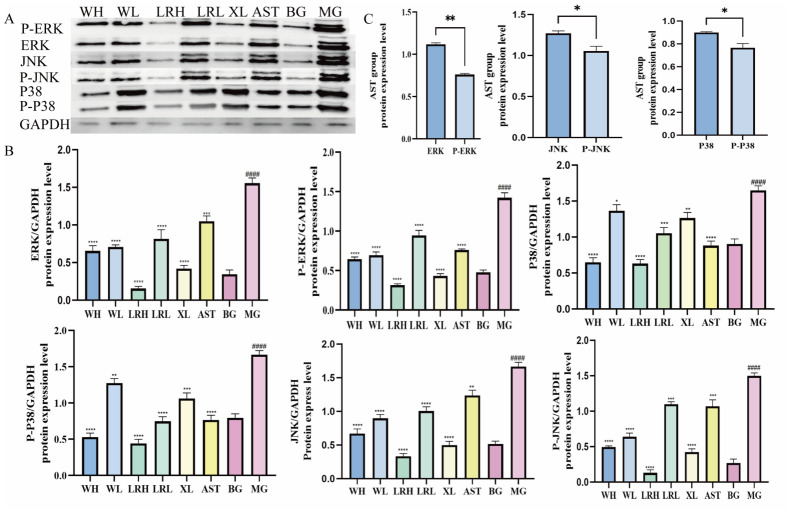
The influence of LR-DBW on the expression levels of MAPK pathway-related proteins in the femoral tissue of rats with osteoporosis caused by RA. (**A**) Western blot test results of protein; (**B**) immunoblotting grayscale analysis; (**C**) AST group protein expression level. (Compared with the BG group: #### *p* < 0.0001; compared with the MG group: * *p* < 0.05, ** *p* < 0.01, *** *p* < 0.001, **** *p* < 0.0001).

The protein expression levels of ERK, JNK and P38 in the rat femur were detected by Western blotting (Figure 7A). The images were analyzed using Image J software (Figure 7B). The results show that compared with the BG group, the expression of the three subtypes of the MAPK pathway, p-ERK, p-JNK and p-p38, was significantly up-regulated in the MG group (*p* < 0.05). Compared with the MG group, all the treatment groups could significantly reduce the protein expression levels of p-ERK, p-JNK and p-P38 in the rat femoral tissue, and the differences were statistically significant (*p* < 0.05). This trend is consistent with the XL group, indicating that the drug may exert its therapeutic effect through the phosphorylation of the ERK, JNK and p38 signaling pathways (Figure 7C).

### 3.8. LR-DBW’s Inhibitory Effect on Osteoclast Differentiation

**Figure 8:**
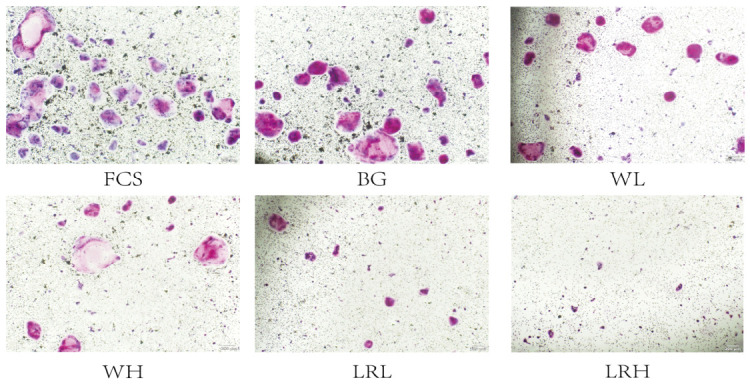
The effect of drug-containing serum on osteoclasts induced by Raw264.7.

The experimental results show that RAW264.7 cells differentiated a large number of osteoclasts under the induction of RANKL (Figure 8). The number of osteoclasts in each group was analyzed and quantified using Image Pro Plus 6.0 software. It was found that with the addition of drug-containing serum, the number of osteoclasts decreased. Compared with the BG group, there was no significant difference in osteoclast differentiation between the FCS group and the BG group. The drug-containing serum had a significant inhibitory effect on osteoclast differentiation (*p* < 0.05), and the inhibitory effect increased with the increase in concentration, with the inhibitory degree being LRH group > WH group > LRL group > WL group. This indicates that LR-DBW can significantly inhibit the differentiation of osteoclasts induced by RANKL.

### 3.9. F-Actin Ring Experiment to Detect the Effect of LR-DBW on the Maturation of Osteoclasts

**Figure 9:**
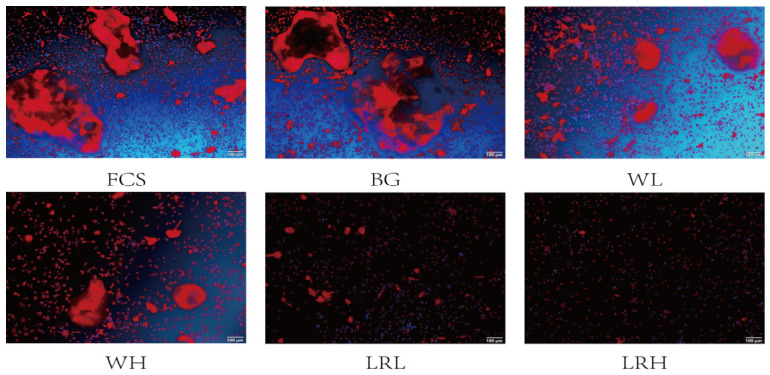
The F-actin ring assay was used to detect the effect of LR-DBW on the actin rings of osteoclasts.

After being induced to differentiate by RANKL, the RAW264.7 cells differentiated into mature osteoclasts and formed actin rings (Figure 9). Subsequently, using Image Pro Plus 6.0 software for analysis, it was found that after treatment with the drug-containing serum, the number and area of actin ring structures in the drug-containing serum group were significantly reduced compared to the BG group (*p* < 0.05). This indicates that fermented deer bone water extract can inhibit the formation of actin rings in mature osteoclasts, and the inhibitory effect is in the order of LRH group > WH group > LRL group > WL group.

### 3.10. The Effect of LR-DBW on the Protein Expression Level of Osteoclasts Induced by RANKL Was Detected by Western Blotting

**Figure 10:**
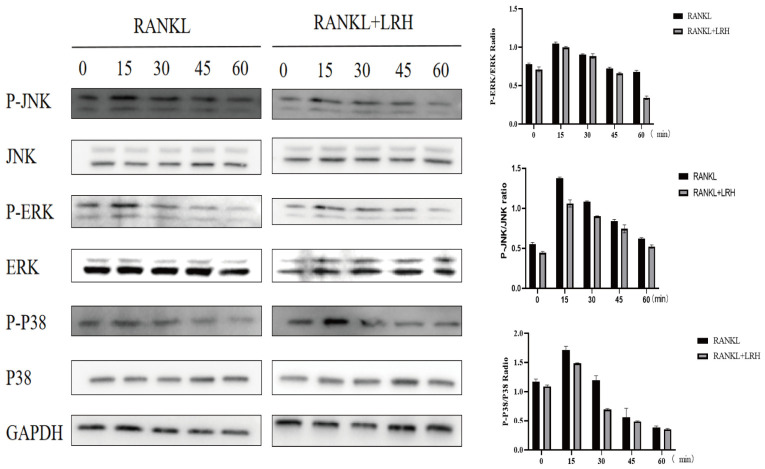
The influence of LR-DBW on key proteins of the MAPK signaling pathway.

In order to better understand the potential mechanism by which LR-DBW inhibits OC differentiation, we evaluated the effects of LR-DBW on the expression of proteins related to the MAPK signaling pathway. The results are shown in Figure 10. After RANKL induction, the phosphorylation levels of JNK, ERK, and p38 increased. However, at 15 min of LR-DBW intervention, the phosphorylation levels of JNK, ERK, and p38 showed a downward trend relative to their total protein levels. These results indicate that LR-DBW may inhibit RANKL-induced osteoclast differentiation by reducing the protein expression levels of p-ERK, p-JNK and p-p38.

## 4. Discussion

This study demonstrates that *Lactobacillus reuteri*-fermented deer bone water extract (LR-DBW) significantly alleviates rheumatoid-arthritis-induced osteoporosis (RA-OP) in a rat model. Our research results indicate that LR-DBW improves bone structure and restores bone metabolic balance by regulating the expression of related proteins in the MAPK signaling pathway. Furthermore, fermentation with *L. reuteri* enhanced the therapeutic efficacy of the deer bone extract compared to the unfermented counterpart. These results provide a scientific basis for developing LR-DBW as a functional food or novel therapeutic agent for managing RA-associated bone loss. Rheumatoid arthritis (RA) is a chronic systemic autoimmune disease characterized by symmetrical, progressive inflammatory polyarthritis [27]. Bone loss, a detrimental consequence of persistent inflammation in RA, significantly increases the risk of fractures and compromises the quality of life [28]. While several pharmacological agents are available for osteoporosis (OP), their long-term use is often limited by adverse effects. Consequently, there is an urgent need to develop safe, non-toxic, and effective alternatives. Deer bone has long been used in traditional medicine for its anti-inflammatory and anti-osteoporotic properties. In this investigation, we utilized *L. reuteri* to ferment deer bone water extract (DBW). *L. reuteri* is a well-documented probiotic with a confirmed safety profile [29,30]. Our preliminary data indicated that *L. reuteri* exhibited rapid growth and notable protease activity in DBW, effectively hydrolyzing proteins into bioactive peptides. This biotransformation likely increased the diversity and bioavailability of peptides in LR-DBW, which is crucial for maintaining bone homeostasis. The types and contents of amino acids in LR-DBW and DBW were determined through amino acid analysis. It was found that the content of histidine was the highest in both LR-DBW and DBW, and the content of histidine in LR-DBW was higher than that in DBW. Current studies have shown that histidine, as an important amino acid in the body, has the effect of promoting calcium absorption and can effectively prevent osteoporosis [31].

To evaluate the therapeutic effects of LR-DBW, we assessed serum biomarkers related to bone metabolism. Parathyroid hormone (PTH) regulates serum Ca^2+^ levels, while calcitonin (CT) acutely inhibits osteoclast activity [32]. Bone Gla protein (BGP, or osteocalcin) and alkaline phosphatase (ALP) are key markers for osteoblast activity and bone formation [33,34,35]. Additionally, MMP-9, a matrix metalloproteinase expressed in mature osteoclasts, reflects osteoclast activity and extracellular matrix degradation. In our study, rats in the model group (MG) exhibited significantly lower serum Ca^2+^ and BGP levels, alongside elevated PTH, CT, ALP and IL-6 levels compared to the blank group (BG). These biochemical alterations, combined with histopathological and Micro-CT observations—such as sparse trabeculae, enlarged marrow cavities, and reduced bone density—confirmed the successful establishment of the RA-OP model. Notably, treatment with LR-DBW, particularly at high doses (LRH), significantly reversed these abnormalities. The LRH group showed increased BV/TV and trabecular number, and decreased trabecular separation compared to the MG group. Moreover, serum Ca^2+^ and BGP levels increased in a dose-dependent manner. The superior efficacy of LR-DBW over unfermented DBW (WH group) suggests that fermentation enhances the bioactive potential of deer bone extract in treating RA-induced OP.

The MAPK signaling pathway serves as a critical hub in eukaryotic cells, translating extracellular stimuli into cellular responses such as proliferation, differentiation, and apoptosis [36,37,38,39]. The pathway comprises four subfamilies: ERK, JNK, p38, and ERK5. In the context of RA-OP, the hyperactivation of ERK, JNK, and p38 drives osteoclastogenesis and inhibits osteoblast function [40,41,42,43]. Specifically, phosphorylated ERK up-regulates core transcription factors like c-Fos and NFATc1, accelerating osteoclast differentiation while suppressing osteogenic factors like Runx2 [44,45,46,47]. Similarly, JNK phosphorylation, triggered by inflammatory stress, activates c-Jun and exacerbates bone microenvironment damage. p38 phosphorylation is essential for osteoclast actin ring formation and the up-regulation of bone-resorbing proteases like MMP-9 [48,49]. Therefore, inhibiting the aberrant phosphorylation of these kinases can restore the balance between bone resorption and formation.

To verify the functions of the relevant proteins, we measured the phosphorylation levels of ERK, JNK and p38 in the femoral tissue. Our results show that the LR-DBW treatment significantly reduced the expressions of p-ERK, p-JNK and p-p38, and the differences were significant compared to the model group. To further verify this mechanism, we used astragaloside IV (AST), which is a known substance that can inhibit the ERK, JNK and p38 signaling pathways [50]. Interestingly, although using LR-DBW alone can effectively inhibit the phosphorylation of ERK, JNK and p38 proteins and improve bone parameters, the combined use of LR-DBW with AST does not produce an additive effect. Compared with the MG group, the AST group showed a significant increase in the number of bone trabeculae, but the effect was not as good as that of the LRH group. This indicates that the bone protection effect of LR-DBW depends on the regulation of key proteins (ERK, JNK, p38) in the MAPK signaling pathway, rather than on excessive inhibition. Although inhibiting the excessive activation of ERK, JNK, and p38 in the inflammation-driven MAPK is necessary to reduce bone resorption, excessive or continuous inhibition may disrupt the dynamic balance of bone remodeling, thereby potentially impairing normal osteoblast function or the rate of bone renewal. Therefore, LR-DBW seems to exert its therapeutic effect by restoring the key proteins (ERK, JNK, and p38) in the MAPK signaling pathway to the physiological baseline level, rather than completely shutting it down.

Consistent with the in vivo findings, our in vitro experiments using RAW264.7 cells confirmed the inhibitory effect of LR-DBW on osteoclastogenesis. TRAP staining revealed that LR-DBW significantly reduced the number of multinucleated TRAP-positive osteoclasts induced by RANKL, with the fermented extract (LRH) showing superior activity compared to the unfermented extract (WH). Furthermore, osteoclast bone resorption is dependent on the formation of the F-actin ring [51,52]. We observed a significant reduction in the number and area of F-actin rings in osteoclasts treated with LR-DBW. This indicates that LR-DBW not only inhibits osteoclast differentiation but also impairs their maturation and resorptive function. The hierarchy of inhibitory effects (LRH > WH > LRL > WL) further supports the notion that fermentation enhances the bioactivity of deer bone extract against osteoclasts. Through protein blotting experiments, it was confirmed that LR-DBW can inhibit the protein expression levels of ERK, JNK and p38. This might be one of the mechanisms by which LR-DBW inhibits the formation of osteoclasts induced by RANKL.

Overall, the results of these in vivo and in vitro studies indicate that the protective effect of LR-DBW on bones may be achieved by regulating the expression of key proteins ERK, JNK, and p38, which provides a mechanistic basis for its potential therapeutic efficacy in treating osteoporosis caused by rheumatoid arthritis.

However, there are complex interactions regarding the mechanism by which LR-DBW alleviates osteoporosis, and the specificity of how the drug exerts its effect through the MAPK signaling pathway still requires further in-depth study in the future.

## 5. Conclusions

In conclusion, this study demonstrates that *Lactobacillus reuteri*-fermented deer bone water extract (LR-DBW) effectively alleviates rheumatoid-arthritis-induced osteoporosis by restoring bone metabolism homeostasis, improving bone microstructure, and suppressing osteoclastogenesis. Inhibiting the excessive activation of ERK, JNK, and p38 may be one of the mechanisms by which LR-DBW treats osteoporosis. Specifically, this is accomplished by reducing the phosphorylation levels of ERK, JNK, and p38, thereby hindering the differentiation of osteoclasts and the formation of actin rings. Notably, fermentation with *L. reuteri* significantly enhanced the bioactive efficacy of deer bone extract compared to the unfermented counterpart. These findings provide a theoretical basis for developing LR-DBW as a promising functional food or dietary supplement for the management of inflammatory bone diseases.

## Figures and Tables

**Table 1:** Joint scoring index.

Score	Standard for Evaluation
0	No redness or swelling.
1	There is slight redness and swelling from the ankle to the area of the tarsal bones.
2	Mild redness and swelling are confined to the metatarsal bones or the ankle (in a single area).
3	Moderate redness and swelling, extending from the ankle joint to the metatarsal area.
4	Severe swelling and redness, extending from the ankle joint to the metatarsal bone, accompanied by joint stiffness and obvious deformity.

## Data Availability

The original contributions presented in this study are included in the article. Further inquiries can be directed to the corresponding authors.

## Abbreviations

The following abbreviations are used in this manuscript:

OP	Osteoporosis
RA	Rheumatoid arthritis
AA	Adjuvant arthritis
LR-DBW	*Lactobacillus reuteri*-fermented deer bone water extract
DBW	Deer bone water extract
OC	Osteoclast
OB	Osteoblast
LR	*Lactobacillus reuteri*
BG	Blank control group
MG	Model group
WH	High-dose group of deer bone water extract
WL	Low-dose group of deer bone water extract
LRH	High-dose group of fermented deer bone water extract
LRL	Low-dose group of fermented deer bone water extract
XL	Positive drug group Xianling Guobao Capsules
AST	Inhibitor group
FCS	Fetal bovine serum control group
Tb.N	Bone trabeculae
Tb.Th	Thickness of bone trabeculae
Tb.Sp	Separation degree of bone trabeculae
BS/BV	Bone surface area/bone volume
CT	Calcitonin
BGP	Bone-γ-Carboxyglutamic Acid-Containing protein
PTH	Parathyroid Hormone
ALP	Alkaline phosphatase
